# Gut microbes regulate the feeding center: a new discovery of Gut Brain Axis

**DOI:** 10.1038/s41392-022-01117-5

**Published:** 2022-08-13

**Authors:** Fengwu Chen, Kaijian Hou, Zhe-Sheng Chen

**Affiliations:** 1 Department of Endocrinology and metabolic diseases, The First Affiliated Hospital of Shantou University Medical College, 515000 Shantou, Guangdong PR China; 2 Department of Pharmaceutical Sciences, College of Pharmacy and Health Sciences, St. John’s University, New York, NY 11439 USA

**Keywords:** Molecular neuroscience, Neurodevelopmental disorders

Recently, Ilana Gabanyi and colleagues published an article in *Science*,^1^ identifying a microbial sensing mechanism that regulates feeding behavior and host metabolism. In their study, a sex- and age-dependent gut-brain cross-talk is revealed. Their study confirmed that hypothalamic neurons can directly sense the structural components of the gut bacterial microbiota and regulate feeding and nesting behavior, affecting food intake and body temperature.

It is known that human behavior is controlled by the brain. In recent years, a growing number of evidence suggests that gut microbiota can affect brain function and host behavior through neural pathways and immune system, forming the microbe-gut-brain axis. There are many potential communications between the gut microbiota and the brain, including immunomodulatory responses, enteroendocrine secretion, liver metabolism, neuronal innervation and signaling from microbial metabolites.^2^ The gut microbiota has the ability to regulate the host’s appetite, food preferences and feeding behaviors through satiety pathways. In addition, it was reposted that the gut microbiota and the brain participated in all the regulation of human activities and behaviors through the process of co-evolution.^3^

The hypothalamus integrates hormonal, sensory and gastrointestinal nutritional signals to regulate feeding behavior. The role of the gut microbiota in host metabolism depends on how it changes hypothalamic activity. Several animal studies suggested that the gut microbiota can change hypothalamic gene expression, levels of neuropeptide and neurotransmitter, and neuronal activity. Although little is known about the exact mechanisms by which the gut microbiota alters hypothalamic physiology, direct effects of microbial metabolism and related microbiota-derived molecules are likely involved. The gut microbiota regulates hypothalamic function may be related to SCFAs (short chain fat acid) and ClpB (a bacterial protein mimetic of α-MSH), that change host feeding behavior through hypothalamic neurons.^2,4,5^

However, it has not been confirmed whether brain neurons can directly sense bacterial components, and whether bacteria can directly regulate the physiological processes of the host by regulating neurons. Since the detailed mechanisms that drive gut microbes to affect brain function and control host feeding behavior are unclear, existing studies based on the effects of microbes on feeding behavior are difficult to be translated into clinical use.

**Fig. 1:**
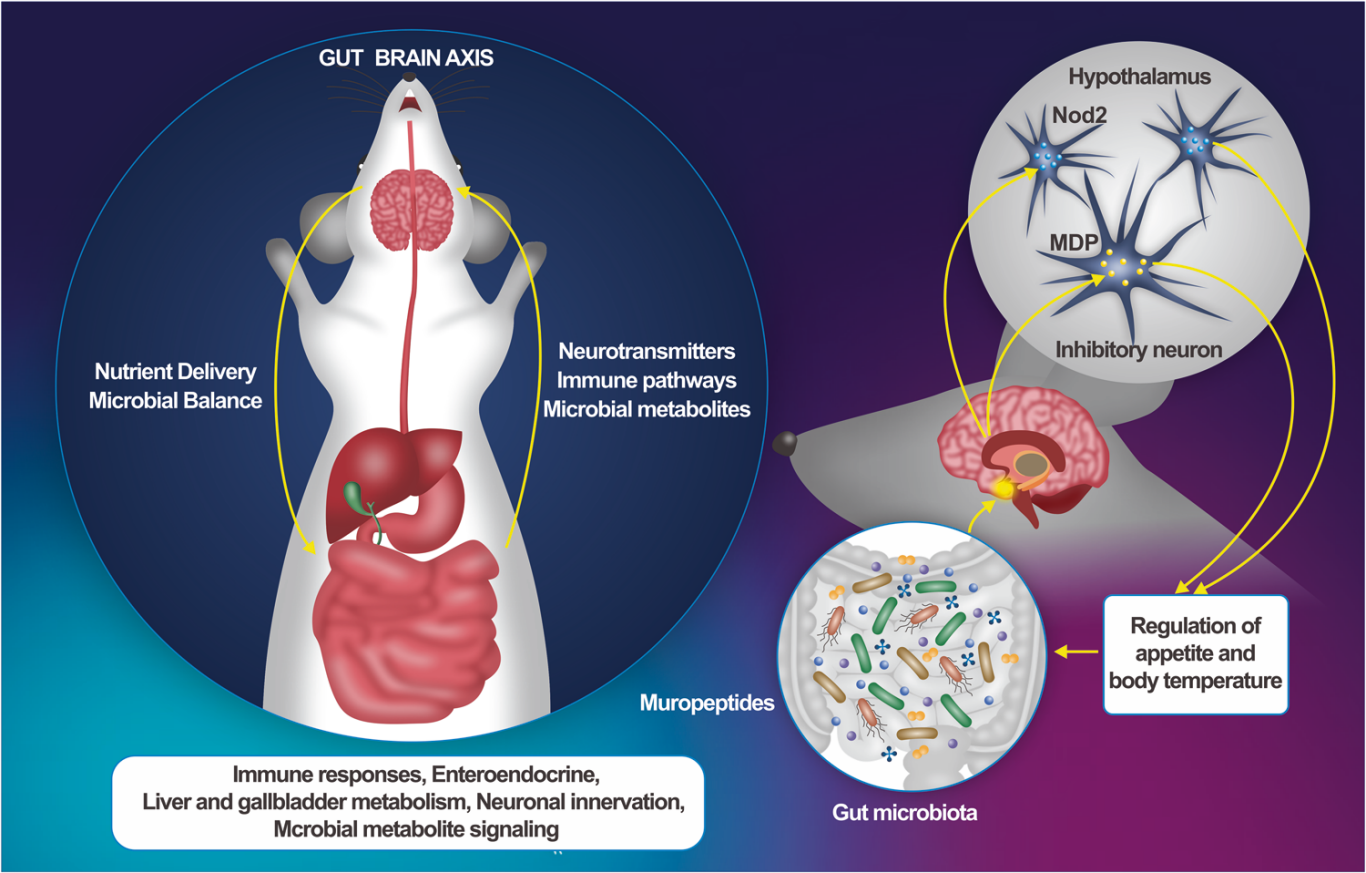
Gut Brain Axis: It is a system composed of the brain and the gut in the body. The brain and the gut work together to regulate the immune responses, enteroendocrine, liver and gallbladder metabolism, neuronal innervation, microbial metabolite signaling (Left). Gut microbes affect feeding behavior by modulating brain function: the microbiota plays an important role in Nod2 ligand production and appetite regulation through Nod2-expressing hypothalamic neurons. The brain can assess food intake and regulation body temperature by sensing changes of gut microbes (Right)

Ilana Gabanyi and colleagues used transgenic mouse and in situ hybridization techniques to confirmed if Nod2, a pattern recognition receptor that recognizes fragments of bacterial cell walls, is expressed in neurons, microglia and endothelial cells. Gavage of radioisotope-labeled MDP (muramyl dipeptide, a ligand of Nod2 in the gut) into the mice confirmed that MDP can reach the brain from the gut, possibly faster in females than in males. When neurons come into contact with bacterial wall peptides in the gut, the electrical activity of neurons is suppressed. MDP treatment caused the mice to eat less. Loss of Nod2 (gene knockout) in inhibitory γ-aminobutyric acid transporter-positive (GABAergic) neurons in aged female mice, these neurons are no longer inhibited by cell wall peptides, and the brain loses its ability to control food intake and body temperature, which increases appetite, render female mice to eat more and gain weight. However, this phenomenon was not found in male mice at the same age. In addition, it can reduce the nesting behavior of female mice and the range of daily body temperature. Moreover, the mice are more likely to develop diabetes and have shorter life span. This behavior is associated with heat retention, response to circadian rhythms, fasting, and adrenaline-stimulated thermoregulation. Therefore, Nod2 receptors expressed by inhibitory neurons play an important role in regulating human metabolism, especially in women. MDP induced different patterns of neuronal activation in young and old female and male mice, with more pronounced effects in older mice. Older female mice showed higher responsiveness to MDP in feeding behavior and thermoregulation, and MDP affected neuronal activation in a sex- and age-dependent manner. Eating and MDP gavage can inhibit GABA neuron activity. MDP increases Vgat^ARC^ neuron activity through Nod2 receptors and affects appetite by modulating hypothalamic circuits. Neuronal excitability was recorded with patch clamp, and it was found that MDP decreases the Vgat^ARC^ neuronal activity in a cell-autonomous manner. Local ablation of Nod2 expression by injecting Cre-expressing virus into the hypothalamus of Nod2flox mice affected body weight and body temperature, indicating that Nod2 expression in hypothalamic neurons is necessary for feeding behavior and thermoregulation in aged female mice. The use of broad-spectrum antibiotics (AXB) resulted in increased body weight in Nod2 flox mice, confirming that microbe derived Nod2 ligands are involved in this regulation. This study demonstrates that the microbiota plays an important role in Nod2 ligand production and appetite regulation through Nod2-expressing hypothalamic neurons. The brain can assess food intake by sensing changes in gut bacteria. Excessive intake of certain foods may stimulate the disproportionate growth of certain bacteria or pathogens, compromising gut microbial balance (Fig. 1).

This study revealed a new gut brain communication pathway, in which gut microbes regulate the feeding behavior of the host to stabilize its gut niche. Understanding the mechanism of gut microbiota affecting host appetite and metabolism will help better understand appetite-related disorders. Regulating the structure and composition of gut microbiota and modulating the feeding activities of appetite center, could be a new research direction for weight control and other metabolic disorders, especially for adult women. This study might lead to new treatments for brain diseases and metabolic diseases such as diabetes and obesity.
